# Design and Verification of Integrated Circuitry for Real-Time Frailty Monitoring

**DOI:** 10.3390/s24010029

**Published:** 2023-12-20

**Authors:** Luis Rodriguez-Cobo, Guillermo Diaz-SanMartin, Jose Francisco Algorri, Carlos Fernandez-Viadero, Jose Miguel Lopez-Higuera, Adolfo Cobo

**Affiliations:** 1 CIBER-BBN, Instituto de Salud Carlos III, 28029 Madrid, Spain; 2 Photonics Engineering Group, Universidad de Cantabria, 39005 Santander, Spain; 3 Department Communications Engineering, University of the Basque Country, 48013 Bilbao, Spain; 4 Instituto de Investigacion Sanitaria Valdecilla (IDIVAL), 39011 Santander, Spain; 5 Psychiatry Service, Marqués de Valdecilla University Hospital, 39011 Santander, Spain

**Keywords:** wireless, load cell, real-time monitoring, sarcopenia, frailty

## Abstract

In this study, a new wireless electronic circuitry to analyze weight distribution was designed and incorporated into a chair to gather data related to common human postures (sitting and standing up). These common actions have a significant impact on various motor capabilities, including gait parameters, fall risk, and information on sarcopenia. The quality of these actions lacks an absolute measurement, and currently, there is no qualitative and objective metric for it. To address this, the designed analyzer introduces variables like Smoothness and Percussion to provide more information and objectify measurements in the assessment of stand-up/sit-down actions. Both the analyzer and the proposed variables offer additional information that can objectify assessments depending on the clinical eye of the physicians.

## 1. Introduction

In recent decades, Europe has experienced noteworthy demographic and epidemiological shifts, introducing unprecedented challenges in the provision of care for the elderly [1]. Existing healthcare systems, designed with a focus on the traditional medical model for isolated acute illnesses, find themselves largely ill-equipped to handle the intricate healthcare requirements of older individuals grappling with frequently persistent multimorbidities, geriatric syndromes, and polypharmacy [2].

While prolonging life is undeniably a crucial objective in public health, the preservation of the innate ability to live independently carries even more profound significance. Disabilities do not just weigh on individuals; they also strain the resilience of healthcare systems [3].

Within this context, the geriatric syndrome of frailty and potential interventions directed at this condition have become notably pertinent [4]. The term "frailty" in the elderly has attracted growing attention, leading to various proposed definitions and assessment tools [5,6]. Despite the diligent efforts of numerous researchers, a universally agreed-upon definition and standardized evaluation methodology remain elusive.

Sarcopenia, characterized by the decline in skeletal muscle mass and strength associated with ageing, emerges as a significant phenomenon in the ageing process and a widely debated subject in the geriatric literature [7,8]. Shifting the discourse toward the repercussions of sarcopenia, such as diminished functional reserve linked to movement capacity, may pave the way for constructing a framework and theoretical structure for the condition. This shift moves beyond mere speculation, providing a response that can be effectively translated into clinical practice. Only through such an approach can we establish a consensus on what requires evaluation and how to assess it. This systematic process is crucial for garnering the approval of regulatory agencies, ensuring that sarcopenia and physical frailty attain clinical recognition as significant conditions and valuable targets for interventions [9].

The awareness of sarcopenia as an outcome of ageing is growing, and its association with an increased risk of adverse outcomes such as falls, fractures, frailty, and mortality is well-established [10]. Several methods have been proposed for assessing muscle mass, strength, and physical performance in clinical trials [11]. While these tools have demonstrated accuracy and reliability in research settings, incorporating many of them into everyday practice poses challenges.

In this current study, we incorporated a novel wireless weight distribution analyzer into a chair to gather pertinent data related to common human postures: sitting and standing up. Given that people engage in this activity multiple times a day, the quality of these actions is intricately linked to various motor capabilities, including speed, other gait parameters, fall risk [3], and even insights into conditions like sarcopenia [5], all of which contribute to a comprehensive understanding of human movement dynamics [2].

The quality of sitting or standing actions is not an absolute measure, and currently, there is no qualitative and objective metric for this parameter. Time is the most crucial variable that can be measured and compared across different samples. However, it is recognized that energy distribution around various body parts can offer an additional layer of valuable information [12].

Numerous tests incorporate a sitting/standing task, particularly in older individuals or specific rehabilitation contexts, often assessing lower body strength or movement [13]. One widely used test is the Timed Up and Go test [14], which involves rising from a chair, walking a set distance, executing a 180-degree turn, returning, and sitting down again, with the time taken being the measured parameter. Another comparable test involves repetitively lifting a chair, and there exists a correlation between the two [9]. These assessments [15,16], and other common tests, could offer useful and valuable insights into functional mobility and strength and may be simple and accessible screening tools for sarcopenia in community-dwelling older adults.

The evaluation of stand-up/sit-down actions in these tests currently relies on the expertise of clinical staff, highlighting the need for new methods to systematically assess these movements. Hence, leveraging the designed analyzer, we introduced distinct variables, namely Smoothness and Percussion, to offer more nuanced insights into the process. These variables aim to objectify measurements and enhance our understanding of the dynamics involved in these actions.

Section 2 describes the main blocks of the electronics design required to obtain the weight distribution analyzer. The subsequent subsections introduce and define two novel parameters, Smoothness and Percussion, designed to objectively assess the dynamics of sitting and standing actions. The mathematical formulations of these parameters are presented, accompanied by insightful graphics for visualization. In Section 3, the experimental setup and results are detailed, showing the potential of the weight distribution analyzer to provide an accurate assessment of physical functionality. This innovative approach, incorporating cost-effective electronics and user-friendly metrics, holds promise for enhancing clinical assessments and the early detection of motor function problems in diverse patient populations.

## 2. Materials and Methods

In the pursuit of user-friendly simplicity, we advocated for employing a routine action: specifically, the act of standing up and sitting on a chair as an indirect indicator of frailty and sarcopenia. The core concept of this study revolved around scrutinizing the temporal dynamics of weight transfer from the chair to the individual’s feet during the process of standing up, assuming the absence of leg-related neurological issues. Similarly, in the sitting process, we delved into the reverse transfer: from the individual’s feet to the chair. This section outlined the fundamental framework of the proposed analyzer and delineated the metrics employed to quantify this intricate process.

### 2.1. Weight Distribution Analyzer

**Figure 1:**
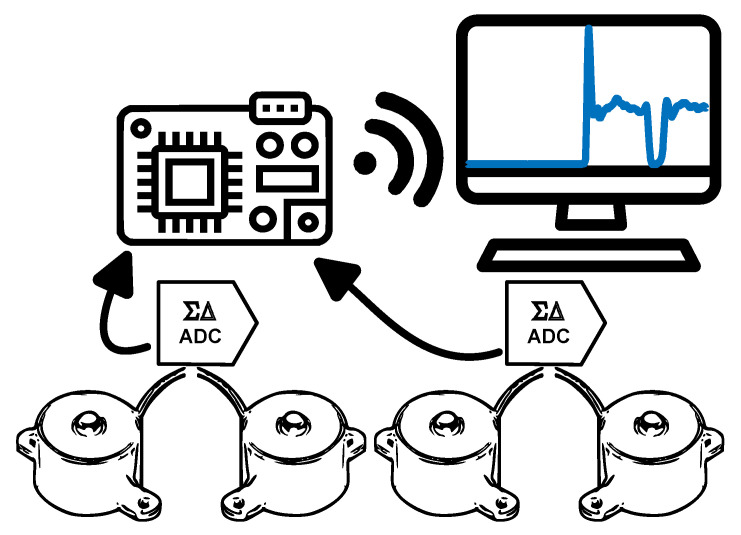
General architecture of the proposed device. Four compression load cells are connected to two precision analog-to-digital converters while being sampled using an ESP32 family microcontroller. This controller sends the data in real time to a desktop computer that displays the measurements.

A chair of a fixed height set at 50 cm and without of armrests was employed for the integration, thereby minimizing the influence of the arms during the analysis to maintain a standardized approach. To capture the weight dynamics, four load cells were integrated into each leg of the chair. Additionally, an electronic system was designed to interrogate these load cells at a high sampling frequency of 1 kHz. The collected data were then transmitted wirelessly to a control computer, facilitating its synchronization with other systems. A schematic representation of the proposed design is illustrated in Figure 1.

As depicted in Figure 1, the system was equipped with four high-precision FC23 compression load cells from TE Connectivity (Schaffhausen, Switzerland), each capable of measuring weights ranging from 0 to 226.80 kg (500 lbs), and which were positioned under every leg of the chair. The Wheatstone bridge signals, amplified by each sensor, were linked to a four-channel precision Delta-Sigma ADC (ADS1219), operating at 1 kHz. With an effective resolution of 20 bits, this ADC utilized the I2C protocol to provide the load values from each sensor.

To manage these data, a generic microcontroller from the ESP32 family by Espressif Systems (China) was employed. This microcontroller, being a low-power system on a chip (SoC) with Wi-Fi and dual-mode Bluetooth capabilities, was a dual-core processor clocked at up to 240 MHz. Its computational prowess and connectivity options enabled the implementation of signal pre-processing algorithms and wireless data transmission.

To ensure data accuracy and minimize noise, the microcontroller calculated the average of three consecutive measures for each reading. The collected data from all legs, as well as their summation, were transmitted via Wi-Fi to an external application at an effective frequency of 50 Hz. The choice of the TCP protocol for communication guaranteed the accurate and orderly transmission of data to their destination.

**Figure 2:**
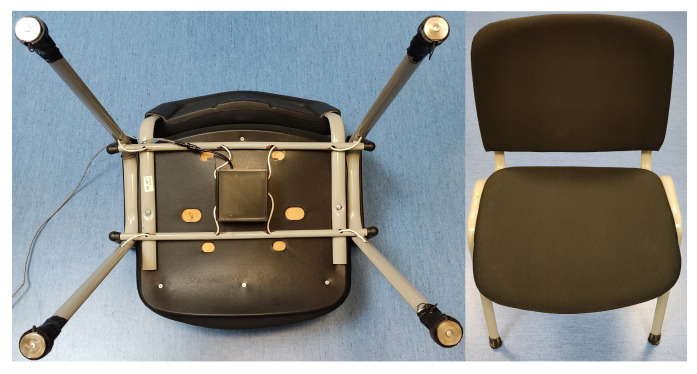
Actual images of the prototype installed on a chair without armrests: the four compression load cells are the only contact points with the floor.

Upon completion of the prototype device, a calibration process involved using weights ranging from 10 kg to 50 kg to fine-tune the response of each load cell. Each device was calibrated following the same procedure. First, the reference value was obtained for each load cell with the empty chair. Then, a 10 kg metal disk was placed in the center of the chair, seeking the most homogeneous weight distribution to obtain the individual coefficient of each cell. Finally, several metallic discs were used to complete the 50 kg load while maintaining their centered position. In this way, the calibration coefficients for each of the cells were confirmed. It is worth mentioning that the differences in calibration coefficients between the different cells varied by less than 2%. The calibrated device then transmitted the aggregated weight data supported by the chair via Wi-Fi at a refresh rate of 50 Hz. This comprehensive setup ensured the precision, reliability, and efficient communication of weight-related information. An image of one of the assembled prototypes is shown in Figure 2.

### 2.2. Passive Parameters

Creating distinct parameters tailored to each action (sitting and standing) provides a precise and objective way to quantify the information gathered by the Smart Chair. The Smoothness parameter can offer insights into the fluidity and ease of the sitting action, while the Percussion parameter can provide a measure of the impact or abruptness associated with the standing action. These parameters not only add specificity to the analysis but also contribute to a comprehensive understanding of the dynamics involved in these routine movements.

#### 2.2.1. Smoothness (S)

Smoothness, S, can be defined as a value that provides information about the body dynamics when it is standing, and it is related to the number of attempts a person makes until they can stand up. Each attempt involves an oscillation in the weight curve, and the magnitude of this oscillation together with the number of attempts generate a value of S. Mathematically, Smoothness can be defined as

(1) $$S=\left\{\begin{array}{cc} 1 & n=0\\ 1-K\cdot C & n>0 \end{array}\right.$$

*K* being a variable that depends on the weight variations in the period prior to incorporation, it has the following form:

(2) $$K=1-\frac{1}{n}\cdot \sum_{i=1}^{n}\left(\frac{x_{i}}{w}\right)$$

where *n* is the number of minimums registered, $x_{i}$ is the value of each one of them, and *w* is the weight registered by the scale when the person is sitting at rest. Finally, *C* is a value that penalizes the number of minimums in the curve so that if there are many important fluctuations, the Smoothness is lesser and has the following form:

(3) $$C=\left\{\begin{array}{cc} 0 & n=1\\ 1+\frac{n}{10}\cdot \left(\frac{1}{K-1}\right) & 1<n\leq 10\\ \frac{1}{K} & n>10 \end{array}\right.$$

**Figure 3:**
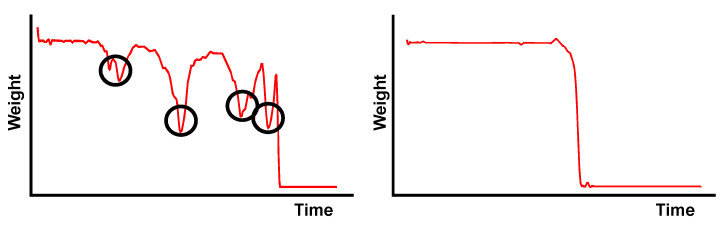
Graphic representation of the Smoothness curves. Pronounced peaks resulting from difficulties in incorporation (**left**). Smooth line without peaks indicating correct incorporation (**right**).

A high Smoothness score, indicating a correct standing action, aligns well with the intuitive understanding of fluid and efficient movements. As depicted in Figure 3 (left), the incorporation of penalties for multiple attempts, especially with a higher threshold like n>0, adds a layer of realism, acknowledging that repeated efforts may indicate difficulty or potential issues with the action.

The consideration that a large minimum is more penalized than a small one, based on the expended energy, is a thoughtful addition. It reflects the understanding that inefficient or difficult attempts should be appropriately accounted for in the assessment. This approach not only encourages correctness in action but also emphasizes the importance of executing it with efficiency and minimal effort.

#### 2.2.2. Percussion (P)

The concept of Percussion (*P*) as the force exerted by the human body during the act of sitting in relation to its force while at rest is a well-founded and insightful parameter. This value essentially captures the rapid increase in force when a person sits down, analogous to the physics concept of great force exerted on an object at an instantaneous moment (F≅inf) with a very small time interval (Δt≈0).

**Figure 4:**
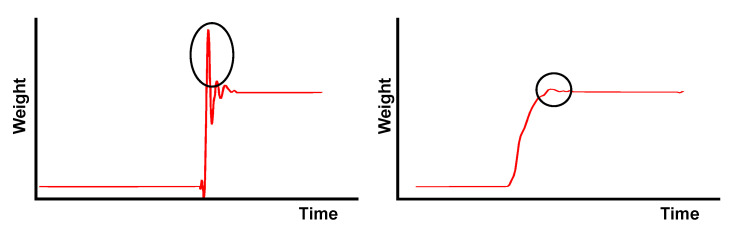
Graphic representation of the Percussion curves. Pronounced peak resulting from difficulties in sitting (**left**). Smooth curve without peaks indicating correct sitting (**right**).

The formulation of Percussion, which relates the relative weight increase between the weight at rest and the weight at the moment the person sits down, adds a quantitative measure to the impact or abruptness associated with the sitting action. This parameter not only offers a means to objectively assess the force dynamics during the sitting process but also aligns with the physical principles underlying the concept of percussion in the context of human movement. A graphical representation of this parameter can be seen in Figure 4.

(4) $$P=1-\frac{w_{0}}{w_{s}}$$

where $w_{0}$ is the weight measured by the chair when the person is sitting at rest, and $w_{s}$ is the weight measured by the chair at the instant the person is down.

The connection between Percussion and low limb force is a logical and insightful correlation. In the case of a healthy individual, the Percussion value would indeed be low, reflecting a smooth and gradual descent into the chair without sudden impacts. On the contrary, if a person struggles to maintain their own weight at any point during the action, resulting in a peak in the weight curve, the Percussion value would be higher. This relationship effectively captures the impact or force exerted during the sitting process.

The defined range of Percussion values, from zero to a limit of one, provides a standardized scale for assessment. The upper limit of one indicates an extreme scenario wherein there is an infinite increase in weight, emphasizing the severity of the impact or force exerted during the sitting action. This parameter not only quantifies the dynamics of the movement but also offers a valuable metric for evaluating the ability to control and manage one’s own weight during the sitting process.

### 2.3. Rise Strategy and Weight Distribution

Rise strategy is a concept based on [17], which evaluates body dynamics during standing up. Two different ways of standing up can be differentiated based on the Base of Separation, BoS, as the distance between the Centre of Mass (CM), that represents the theoretical point where the entire mass is considered to be concentrated, and the foot position.

**Figure 5:**
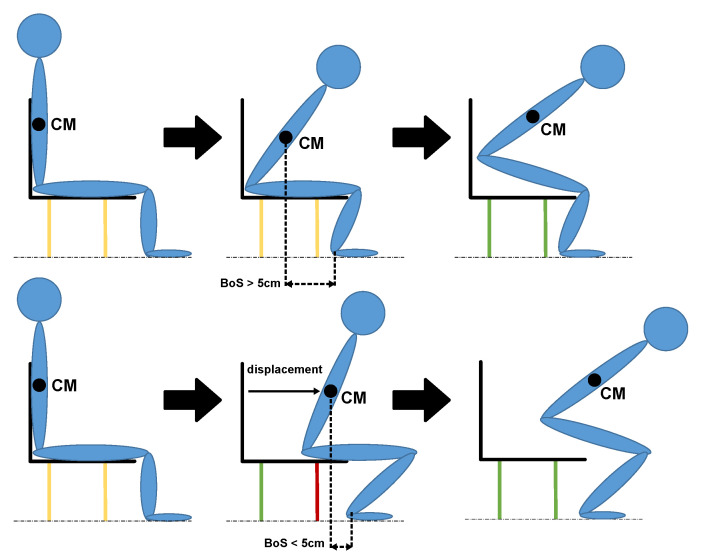
Graphic representation of rise strategy. Phases of “momentum transfer” strategy, in which BoS must be greater than 5 cm (**top**). Phases of “stabilization” strategy, in which BoS must be less than 5 cm (**bottom**).

The first is a fast incorporation, called “momentum transfer”, in which the center of mass of the body, CM, begins to rise from the beginning and in which the feet are kept almost fixed. This phase is characterized by a BoS greater than 5 cm and a center of mass speed of more than 10 cm/s. The second is a slower method, called “stabilization”, and requires a phase of preparation in which the center of mass and the feet are brought closer together prior to incorporation. This phase is characterized by a BoS of less than 5 cm and a center of mass speed of less than 7.5 cm/s. If a strategy does not meet the conditions of any of the above, then it is called “combined”. A graphical representation of both rising strategies is depicted in Figure 5.

## 3. Experimental Setup and Results

The proposed device was installed at the Photonics Engineering Group facilities at the University of Cantabria. Leaving a free space of several meters in front of the chair, the chair was placed together with some marks on the floor in order to perform a reference test for the analysis of frailty: Timed up and Go (TUG) [14]. The TUG test consists of measuring the time it takes a person to stand up from a chair, walk a few meters (3–4 m) at his or her usual pace, turn around, return to the chair, and sit down.

Conducting a study with volunteers without mobility problems to establish baseline values for the developed metrics and comparing them with the TUG test time is a prudent step. This not only gauges the usability of the device but also provides a benchmark for understanding how well the proposed metrics align with established measures of mobility.

Furthermore, the execution of specific tests to explore the detection extremes of the defined variables adds depth to the evaluation process. This allows for a thorough examination of the device’s capabilities and its ability to capture a wide range of scenarios, contributing to the overall robustness and reliability of the system.

### 3.1. Smoothness and Percussion Evaluation

The device was used to evaluate the standing up and sitting down actions of 15 healthy volunteers (13 men and 2 women) within the TUG test, whose characteristics are summarized in Table 1.

All the tests performed by the volunteers offer baseline parameters in accordance with the variable definitions, as expected. The detailed insights provided by Percussion and Smoothness variables demonstrate their potential for complementing traditional measures like total time in the Timed Up and Go test.

The granularity offered by Percussion, particularly in capturing the impact of sitting back on the chair, adds valuable information to the assessment. Additionally, Smoothness acts as a sensitive indicator, raising alerts when an individual’s mobility is significantly impaired, which is made evident by the need for multiple attempts to stand up.

**Figure 6:**
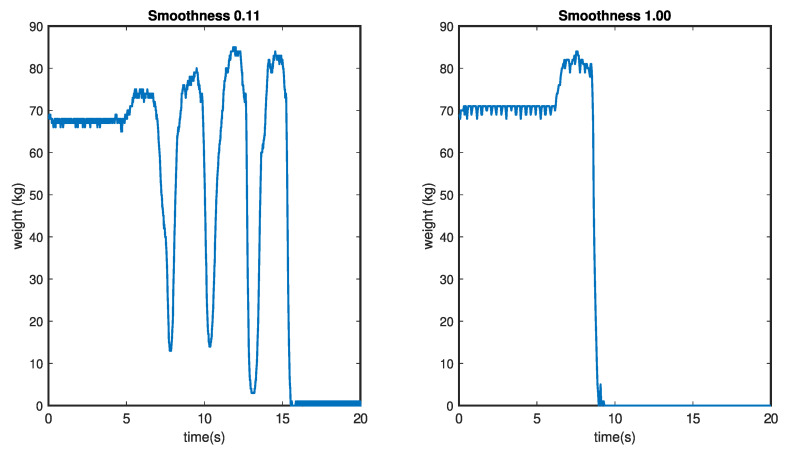
Simulation of weight change during the stand-up of a person with reduced mobility. The measured Smoothness for this change is S=0.11, which corresponds to a simulation in which a person makes 3 unsuccessful attempts before succeeding in standing.

The specific laboratory tests simulating scenarios wherein individuals require multiple attempts to stand up provide a valuable validation of the system’s capabilities. The results, as depicted in Figure 6, likely offer a clear visualization of the challenges faced by individuals with compromised mobility and emphasize the sensitivity of the Smoothness parameter in detecting such scenarios.

**Figure 7:**
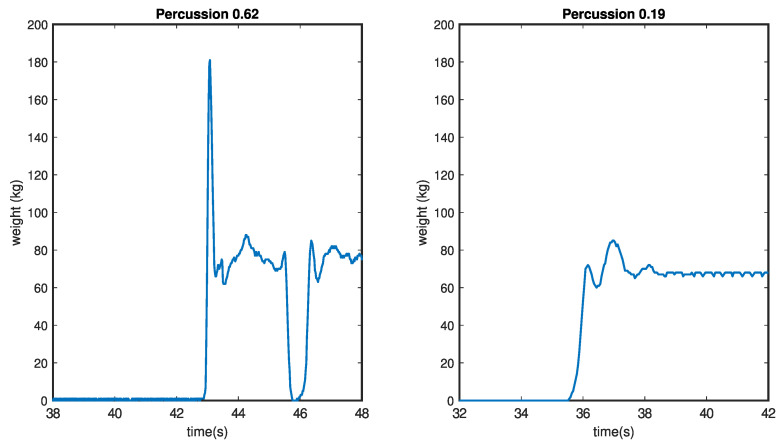
Simulation of weight change during the sit-down of a person with reduced mobility. The measured Percussion for this change is S=0.62, which corresponds to an increase in weight during sitting of 2.5 times the static value.

Laboratory tests have also simulated situations in which the patient does not correctly control the action of sitting back in the chair. A typical situation for people with mobility problems is that they drop their body when sitting down, causing a sudden impact against the chair. The Percussion variable reflects this situation by offering a value higher than the reference values obtained in the initial tests. This situation is exhibited in Figure 7.

### 3.2. Rise Strategy Evaluation

The developed device allows us to obtain the weight distributed on each of the legs. Therefore, it is possible to isolate the load balance between the front and rear legs, allowing us to identify the incorporation strategy. Below are some of the results of the specific tests when showing the separate load on the front and rear of the chair.

**Figure 8:**
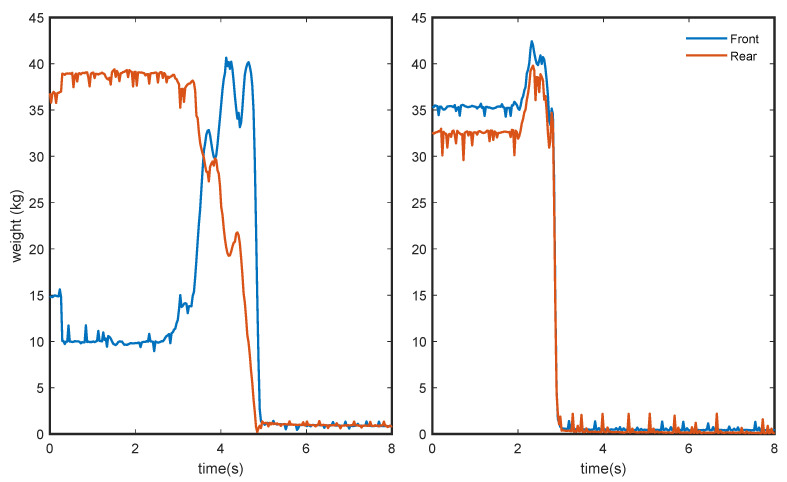
Simulation of weight change during the stand-up of a person while varying the standing strategy: by shifting the weight forward (“Stabilization”, **left**) and by directly rising from the beginning (“Momentum transfer”, **right**).

Figure 8 depicts the chair response developed when both stand-up strategies were simulated. In particular, when the patient’s body mass shifts from the rear legs to the front legs before starting to unload the chair, it can be considered a “stabilization” (Figure 8, left). On the contrary, when both parts of the chair, front and rear legs, are unloaded simultaneously, we can consider that stand-up is performed directly, according to the “momentum transfer” strategy (Figure 8, right).

## 4. Discussion

Building upon this platform, two new variables, Smoothness and Percussion, were introduced. These variables aim to provide objective and complementary values to those conventionally employed in clinical assessments. Laboratory testing underlines the efficacy of both the device and the defined variables, generating reference values for healthy individuals and exploring scenarios wherein motor function is compromised. The results suggest that this approach offers enhanced granularity in quantifying standard clinical tests, such as the Timed-Up-and-Go timing test. Overall, this device and the associated variables exhibit promise in providing nuanced insights into the physical functionality of individuals.

Since the device can provide the independent load value for each leg, separating the loads applied to the front and rear area of the chair, it is possible to analyze the time evolution of the weight shift (if any). This measurement gives us additional information on the stand-up process, allowing us to determine the strategy used. This information can be of great help in establishing clinical thresholds for the early identification of motor function problems, muscle strength, and its possible relationship with clinical sarcopenia [18].

In anticipation of advancing the understanding and assessment of elderly individuals’ physical functionality, this study introduced a pioneering approach with the weight distribution analyzer, aiming to address the complexities of frailty and sarcopenia. The proposed findings included an enhanced view of routine movements through the novel parameters, Smoothness and Percussion. The actual findings, as detailed in the experimental setup and results section, demonstrate the device’s efficacy in capturing weight distribution dynamics during sitting and standing actions. The introduced parameters offer granularity in assessing mobility, with the Smoothness metric effectively detecting difficulties in standing, and Percussion quantifying the impact during sitting. These outcomes not only align with the initial objectives but also signify a potential paradigm shift in clinical assessments. The Smart Chair, with its user-friendly design and insightful metrics, showcases promise in enhancing patient monitoring and contributing to the early identification of motor function problems and sarcopenia.

## 5. Conclusions

Aligned with some of the many works that employ new information and communication technologies to monitor lifestyles [19] and encourage active habits [20], this work introduces an innovative and non-invasive approach to assess the physical functionality of patients, utilizing cost-effective electronics to capture reliable and repetitive data from a common daily activity: sitting down and standing up from a chair. The device, designed to instrument each leg of a standard armrest-free chair with load cells, incorporates a 32-bit microcontroller with WiFi communication capability. This setup enables the real-time monitoring of the weight supported by the chair, allowing for the extraction of diverse weight transfer profiles during the sitting and standing actions.

In summary, this study presents a user-friendly device designed for seamless deployment in clinical environments. The simplicity of both the device and the defined variables facilitates straightforward integration into the clinical monitoring of patients with diverse pathologies, including existing tests like the Timed-Up-and-Go. This potential clinical integration holds promise for enhancing the objective information accessible for assessments, particularly in complex processes like motor function. The ease of use and potential for early detection offered by this approach could significantly improve patient monitoring, providing valuable insights and contributing to the timely identification of critical situations.

Given that the primary aim of this study is to validate the weight analyzer technology for its prospective clinical application, it is crucial to acknowledge certain limitations inherent to this initial validation phase. The selected sample comprised 15 healthy volunteers without apparent mobility issues, facilitating the acquisition of preliminary data. However, this choice poses limitations in terms of diversity and clinical representativeness. The intention was to establish a baseline for detection, observing subtle variations among participants in controlled conditions. It is noteworthy that clinical application will necessitate additional validations with a broader and more diverse sample, including individuals with varying mobility conditions and advanced age groups. Furthermore, short-term evaluation in a laboratory setting may not fully capture the efficacy of the weight analyzer in real-world scenarios, where external factors could influence its performance. These limitations should be considered in future research to ensure a successful transition of the technology to more complex and varied clinical environments. 

## Figures and Tables

**Table 1:** Characteristics of 15 volunteers. * Body Mass Index. Percussion and Smoothness are compared to the total time spent during the Timed Up and Go test.

Age (Years)	Weight (Kg)	Height (m)	* BMI (Kg/m${}^{2}$)	P	S	TUG Time (s)
28	66	1.75	21.55	0.23	1	8.2
31	83	1.82	25.05	0.11	1	9.8
38	74	1.6	28.9	0.25	1	11.8
32	60	1.73	20.04	0.12	1	9.33
44	93	1.78	29.35	0.06	1	9.27
50	64	1.7	22.14	0.31	1	7.03
48	106	1.82	32	0.1	1	9
24	74	1.83	22.09	0.16	1	8.8
25	77	1.78	24.3	0.04	1	9.3
37	95	1.84	28.06	0.09	1	8.03
30	75	1.68	26.57	0.26	1	6.47
34	100	1.84	29.53	0.2	1	7.83
28	72	1.65	26.44	0.13	1	7.73
26	81	1.72	27.37	0.15	1	9.77
69	90	1.69	31.51	0.08	1	10.07

## Data Availability

The datasets generated and/or analyzed during the current study are available from the corresponding author upon reasonable request.
